# Response of the Arctic Pteropod *Limacina helicina* to Projected Future Environmental Conditions

**DOI:** 10.1371/journal.pone.0011362

**Published:** 2010-06-29

**Authors:** Steeve Comeau, Ross Jeffree, Jean-Louis Teyssié, Jean-Pierre Gattuso

**Affiliations:** 1 Centre National de la Recherche Scientifique-Institut National des Sciences de l'Univers, Laboratoire d'Océanographie de Villefranche, Villefranche-sur-Mer, France; 2 Université Pierre et Marie Curie-Paris 6, Observatoire Océanologique de Villefranche, Villefranche-sur-Mer, France; 3 Marine Environmental Laboratories, International Atomic Energy Agency, Monaco, Principality of Monaco; Paleontological Institute, Russian Federation

## Abstract

Thecosome pteropods (pelagic mollusks) can play a key role in the food web of various marine ecosystems. They are a food source for zooplankton or higher predators such as fishes, whales and birds that is particularly important in high latitude areas. Since they harbor a highly soluble aragonitic shell, they could be very sensitive to ocean acidification driven by the increase of anthropogenic CO_2_ emissions. The effect of changes in the seawater chemistry was investigated on *Limacina helicina*, a key species of Arctic pelagic ecosystems. Individuals were kept in the laboratory under controlled pCO_2_ levels of 280, 380, 550, 760 and 1020 µatm and at control (0°C) and elevated (4°C) temperatures. The respiration rate was unaffected by pCO_2_ at control temperature, but significantly increased as a function of the pCO_2_ level at elevated temperature. pCO_2_ had no effect on the gut clearance rate at either temperature. Precipitation of CaCO_3_, measured as the incorporation of ^45^Ca, significantly declined as a function of pCO_2_ at both temperatures. The decrease in calcium carbonate precipitation was highly correlated to the aragonite saturation state. Even though this study demonstrates that pteropods are able to precipitate calcium carbonate at low aragonite saturation state, the results support the current concern for the future of Arctic pteropods, as the production of their shell appears to be very sensitive to decreased pH. A decline of pteropod populations would likely cause dramatic changes to various pelagic ecosystems.

## Introduction

The oceans have absorbed about one quarter of anthropogenic CO_2_ emissions since 1800 [1], generating profound changes in the ocean carbonate chemistry [2]. Among these changes is a decrease of surface ocean pH; hence the overall process is often referred to as “ocean acidification”. The decrease in pH is already measurable and the global mean decrease is about 0.1 unit since the end of 18^th^ century. According to model projections, the global mean surface pH will decrease by another 0.3 unit by the end of the present century [3]. Some effects of ocean acidification are already detectable. For example, the shell weight of foraminifera from the Southern Ocean has declined by 30 to 35% since the end of the 18^th^ century [4]. However, the consequences of future changes in the carbonate chemistry remain uncertain in many taxonomic groups. Several studies on phytoplankton [e.g. 5], commercial mollusks [6], corals and coralline algae [e.g. 7]–[9] point to a decrease of calcification with a decline in seawater pH. On the other hand, a smaller number of studies [e.g. 10] have shown that the calcification rate of some organisms is unaffected or enhanced at lower pH.

Due to their specific physical and chemical characteristics, the polar oceans are highly vulnerable to ocean acidification and will be the first to face undersaturation with respect to aragonite, a metastable form of calcium carbonate more soluble than calcite in seawater [11]. Model projections suggest that polar surface waters will become undersaturated with respect to aragonite in 2050 in the Southern Ocean [12], [13] and as early as 2016 during one month per year in the Arctic Ocean [14]. In fact, undersaturation has been documented in the Canada basin of the Arctic ocean [15]. Despite the high vulnerability of polar areas to ocean acidification, the biological, ecological and biogeochemical consequences are not well documented.

One of the key organisms of polar pelagic ecosystems is the shelled pteropod, *Limacina helicina*. Pteropods are strict pelagic mollusks that are highly adapted to life in the open ocean. They are commonly referred to as “sea butterflies”, due to their wing-like parapodia evolved from the original gastropod foot [16]. They produce large mucus webs to filter-feed on phytoplankton but also small zooplankton or their own juveniles [17]–[19]. Species of the Order Thecosomata produce a fragile external calcium carbonate shell, which could serve as a ballast enabling large vertical migrations and as a protection against predators. The aragonitic composition of the shell makes it very sensitive to dissolution. *Limacina helicina* is the only thecosome pteropod in Arctic waters. In contrast to the accepted view, it has recently been shown that its distribution is not bipolar as Arctic and Antarctic individuals belong to two genetically distinct species [20]. *L. helicina* plays an important role in the marine food web as a major dietary component for predators such as large zooplankton, herring, salmon, whales and birds [21]–[24]. Shelled pteropods also play a geochemical role in the oceans, as they contribute to the export of calcium carbonate [25] and can represent a major component of the carbon transport to the deep ocean [26], [27].

The response of pteropods to ocean acidification under controlled experimental conditions has been investigated in a single study [28] at two pCO_2_ levels. They exhibited a 28% decrease of calcification when exposed to a pH value predicted in 2100. But their response to separate and combined effects of temperature and pH has not yet been studied. However, Reynaud et al. [9] and Rodolfo-Metalpa et al. [29] have shown that the rate of calcification of corals and bryozoans is not affected by elevated pCO_2_ alone but can be affected by elevated temperature alone or by a combined increase of pCO_2_ and temperature. In the present study, the individual and combined effects of two temperatures (control ∼0°C and high ∼4°C) and five pCO_2_ levels (280, 380, 550, 760 and 1120 µatm) are investigated in *Limacina helicina*. The pCO_2_ levels were chosen in order to cover a range from the preindustrial values to the 2100 worst-case projection (A1FI) of the Intergovernmental Panel on Climate Change (IPCC). The calcium carbonate precipitation rate was estimated as the incorporation of ^45^Ca, and the respiration and gut clearance rates were also measured under these conditions to characterize their physiological status.

## Results

### Sampling, maintenance of organisms and carbonate chemistry

The freezing condition of the fjord delayed the collection of pteropods until 15 May 2009, when they were found at approximately 15 m depth. The size of the organisms present in the fjord, measured as the maximum diameter of the shell, ranged between 1 and 4 mm, indicating that the pteropods were juveniles or males [19]. Animals from the same size range (3 to 4 mm) were used during the experiments.

The parameters of the seawater carbonate system measured in the field (Table S1) showed a low pCO_2_, which increased from 17 May to 2 June (169 to 257 µatm). Supporting information (Tables S2 and S3) provide the average carbonate chemistry parameters in the culture tanks during measurements of respiration and gut clearance, and ^45^Ca uptake. pH exhibited small changes during the course of the experiments with standard deviations ranging from 0.01 to 0.04. Temperature was maintained at 0.5±0.1°C (CT) and 3.9±0.1°C (HT) during the ^45^Ca incubations and at 0.3±0.1°C (CT) and 3.8±0.2°C (HT) during measurements of respiration and gut clearance. Note that the pteropods were exposed to undersaturated conditions with respect to aragonite in the experimental combinations CT 1120, HT 1120, CT 760 and HT 760 (Ω_a_ = 0.57, 0.64, 0.76 and 0.91). In all experiments, including in the 3 d long measurements of the gut clearance, the survival rates for pteropods were 100%, suggesting that the pteropods did not experience an excessive level of stress from sampling and during experimental exposures.

### Size weight relationship, respiration and gut clearance

The size weight relationship (Figure S1) follows an allometric relationship 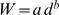, with *a* = 0.23±0.02 (mean ± SD, p<0.001, n = 70) and *b* = 3.04±0.08 (p<0.001, n = 70). The allometric coefficient *b* is not significantly different from the typical value accepted (*b* = 3). This relationship was used to determine the weight of the pteropods used to measure the respiration and gut clearance.

**Figure 1:**
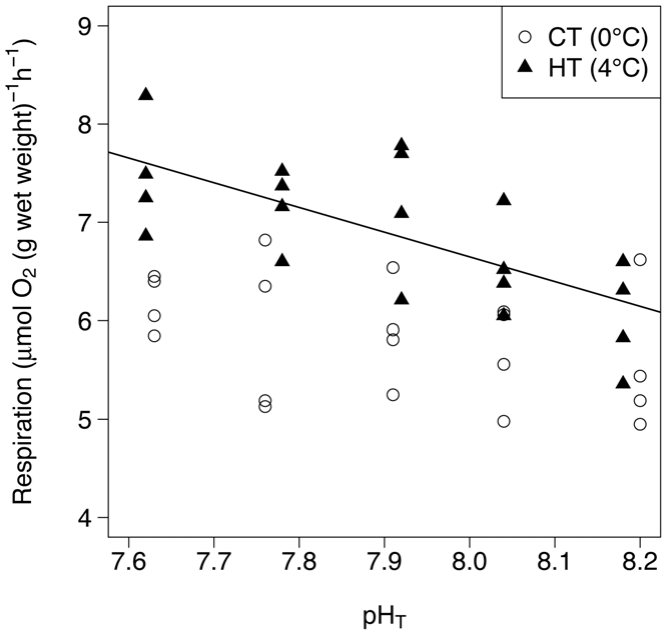
Respiration rate measured at the 5 pCO_2_ levels and at control (CT) and elevated (HT) temperatures. The regression line for the elevated temperature condition is shown (*y* = 26.7−2.5 *x*; R^2^ = 0.47; n = 20).

Results of the respiration rate measurements in the 5 pCO_2_ conditions at control and high temperature are shown in Figure 1 and the mean ± SD respiration rates are presented in supporting information (Table S4). The respiration rate is significantly higher at high temperature than at control temperature (ANOVA, F_1, 38_ = 46, p<0.001). There is no significant effect of pCO_2_ on respiration measured at the control temperature (ANOVA, F_4, 15_ = 0.57, p = 0.69) whereas the effect is statistically significant at high temperature (ANOVA, F_4, 15_ = 3.93, p<0.05). The respiration rate increases by 0.25±0.06 µmol O_2_ (g wet weight)^−1^ h^−1^ for each 0.1 pH unit decrease. The coefficient of determination (R^2^) of the respiration rates at high temperature regressed against time is 0.47. The mean *Q*_10_ value derived from measurements made at the two temperatures is 1.6±0.2.

**Figure 2:**
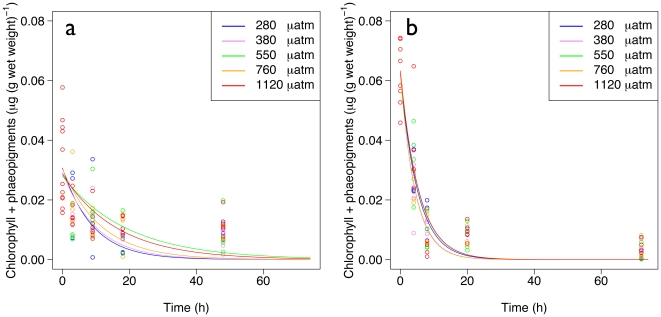
Pigment content in the gut as a function of time. Pigment content at control (CT∼0°C, panel *a*) and elevated (HT∼4°C, panel *b*) temperatures are shown.

The content of chlorophyll and phaeopigments in the gut decreases with time at all pCO_2_ conditions and at the two temperatures (Figure 2). After linearization, a one way analysis of variance confirmed that at both temperatures there is no significant effect of pCO_2_ on the gut clearance rate (F_4, 146_ = 0.53, p = 0.71 and F_4, 116_ = 1.41, p = 0.23, respectively at CT and HT). The mean initial gut content (*G_0_*) and mean gut clearance coefficient (*k*) is respectively 0.03±0.01 µg (g wet weight)^−1^ and 0.08±0.02 h^−1^ at control temperature, and 0.06±0.01 µg (g wet weight)^−1^ and 0.20±0.02 h^−1^ at elevated temperature. The gut clearance *Q*_10_ between the control and elevated temperatures is 12.96.

### ^45^Ca uptake

**Figure 3:**
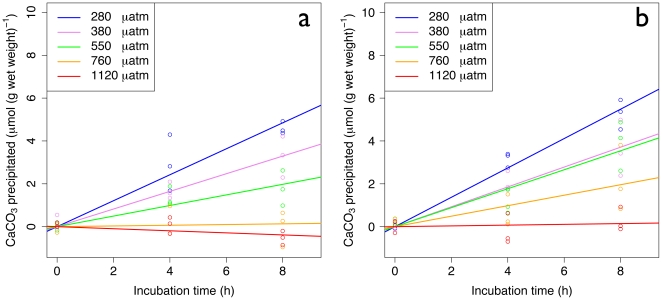
Calcium carbonate precipitation based on ^45^Ca uptake as a function of time. CaCO_3_ precipitation at control (CT∼0°C, panel *a*) and elevated (HT∼4°C, panel *b*) temperatures are shown.

^45^Ca incorporation as a function of time is linear at both temperatures (Figures 3a and 3b). Analysis of covariance (ANCOVA) shows a highly significant effect of pCO_2_ on the rate of calcification at the control (*F*_4, 40_ = 13.105, p<0.001) and elevated temperature (*F*_4, 40_ = 6.538, p<0.001). The highest rates occurred at preindustrial pCO_2_ (280 µatm). At both temperatures calcification decreased as a function of increasing pCO_2_. At CT and at the higher pCO_2_ (760 and 1120 µatm), the pteropods did not incorporate any ^45^Ca (the calcification rate was 0). In contrast, at HT, ^45^Ca incorporation occurred at 760 µatm and was only inhibited at 1120 µatm. The aragonite saturation state was below 1 in the three conditions where no ^45^Ca uptake occurred (Ω_a_ = 0.58, 0.63 and 0.78). All rates of CaCO_3_ precipitation are shown in supporting information (Table S5).

**Figure 4:**
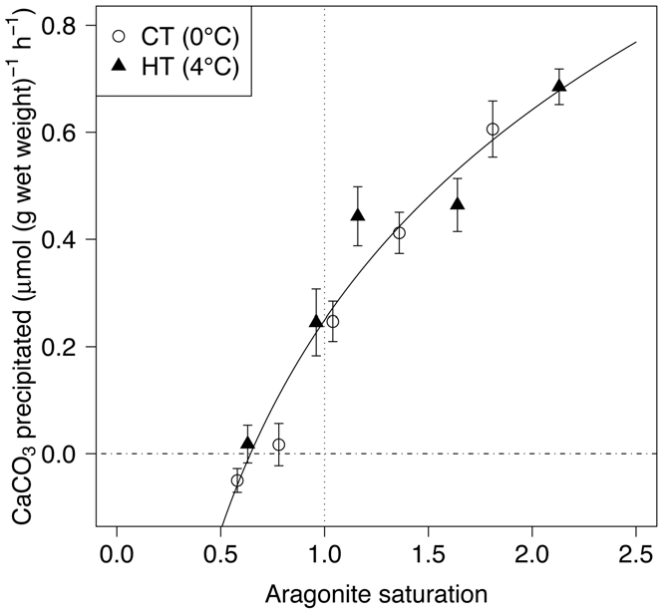
Calcium carbonate precipitated as a function of the aragonite saturation state. The logarithmic regression line is shown.

The rate of calcification is highly correlated with the saturation state of aragonite (Ω_a_) at both temperatures (Figure 4). The fitted model was 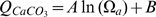, where *A* = 0.57±0.04 and *B* = 0.25±0.02. The coefficient of determination (R^2^) is 0.95. This relationship suggests that calcium carbonate precipitation could occur until Ω_a_ is about 0.64.

## Discussion

The Arctic Ocean and the ecosystems that it hosts are particularly vulnerable to the impact of seawater chemistry changes associated with enhanced atmospheric CO_2_ levels and resulting ocean acidification. An undersaturation with respect to aragonite for at least one month per year in 10% of the Arctic is predicted to occur by the end of the present decade [14]. During our sampling, Kongsfjorden waters were oversaturated with respect to aragonite, with a mean Ω_a_ of 2.23. The lowest Ω_a_ value (1.88) was measured at the end of the experiment (2009-06-02), suggesting that values close to 1 could be reached in the late summer. Kongsfjorden is exposed to the influence of water masses from the north Atlantic or the Arctic depending on season and year [30]. The low values of temperature and total alkalinity measured in surface samples were probably related to a strong influence of Arctic waters. The low pCO_2_ values measured in the fjord were probably due to photosynthetic CO_2_ uptake during the phytoplankton bloom that follows the ice retreat.

This study is the first to provide information on the response of pteropod respiration to a wide range of pCO_2_ conditions. Seibel et al. [31] have reported respiration rate of 3.8 to 6.4 µmol O_2_ (g wet weight)^−1^ h^−1^ for *Limacina helicina antarctica* depending on temperature and pteropod activity. These values are within the range of those measured in the present study (5.6 to 7.5 µmol O_2_ (g wet weight)^−1^ h^−1^). Respiration was the only physiological parameter responding to a combined effect of elevated pCO_2_ and temperature. It increased with increasing pCO_2_ at elevated temperature, demonstrating an increase in metabolic activity in response to the combined effects of pCO_2_ and temperature. In contrast, Fabry et al. [32] reported a 25% decrease in the respiration of *L. helicina antarctica* at −1.86°C and 789 µatm compared to the control (of unspecified pCO_2_). The discrepancy between the two studies may be due by the temperature difference (∼6°C) and the possible different physiology between the two species [20]. Our results were not caused by a stress resulting from sampling or from the experimental conditions as pteropods were pre-acclimated to the experimental conditions for 24 h before the beginning of the incubation, all individuals were active and no mortality occurred. The *Q*_10_ value derived from measurements made at the two temperatures (1.60±0.2) is similar to the range of values reported for zooplankton (1.63 to 1.89, [33]). Future studies might be performed on anesthetized organisms in order to estimate the basal respiration rates.

With regard to gut clearance rates, *L. helicina* appears to be resilient to the different pCO_2_ levels investigated which do not seem to affect metabolic and enzymatic functions. Bernard and Froneman [34] have measured, using the same method as the one used in the present study, a gut clearance rate *k* of 1.33 h^−1^ for the sub-Antarctic *Limacina retroversa*. This value is higher than the one observed in our study (0.082 and 0.201 h^−1^, respectively at CT and HT). This difference might be explained by several factors such as different initial gut content, species and/or temperature. Gut clearance rates consistent with the ones measured in the present study have been reported in other zooplanktonic organisms such as the copepod *Calanus simillimus* (*k* = 0.32 h^−1^, [34]). *Q*_10_ value of 2.21 has been reported in copepods when food is not limiting [35]. The *Q*_10_ estimate of 13 found in the present study is not reliable because the initial gut content of the field population sampled for the experiment at elevated temperature was 113% greater than in the population sampled for the experiment at control temperature. Consequently, *Q*_10_ is greatly overestimated.

In contrast with some previous studies [9], no synergistic effect of elevated pCO_2_ and temperature was found on the precipitation of calcium carbonate. Also, a decrease of incorporation after a few hours of incubation reported in previous studies performed on pteropods [28], [36] was not found. The incorporation of calcium carbonate was linear throughout the 8 h incubation, perhaps because the pteropods were pre-acclimated for 24 h prior to the measurements. The difference could also be due to different animal sizes. Comeau et al. [28] used much larger individuals, that are comparatively more difficult to maintain in the laboratory, than in the present study. Fabry et al. [32] have previously reported a decrease of calcification as a function of increasing pCO_2_ but the seawater carbonate chemistry was not reported, preventing a comparison with the present study.

The relationship between the rate of calcification and the aragonite saturation state was best described by a logarithmic function. A linear regression provides an *R* ^2^ that is only marginally smaller than that of the logarithmic function (0.92 vs. 0.95) but overestimates the precipitation rate at Ω_a_<1. Langdon and Atkinson [8] have proposed a linear parameterization to describe the relationship between coral calcification and Ω_a_ which suggests that calcification does not occur when Ω_a_ is below 1. Other models of the impact of ocean acidification on calcification usually propose that no calcification occurs in waters undersaturated with respect to calcium carbonate [37], [38] but our data demonstrate that *L. helicina* can precipitate calcium carbonate at Ω_a_ values well below 1. In contrast to the general opinion that Arctic pteropods are greatly threatened by ocean acidification, *L. helicina* seems to be relatively more resilient to elevated pCO_2_ than other aragonitic organisms such as corals. This new result would probably needs to be taken into account in biocheochemical models, for example models of the future export flux of aragonite [e. g. 39].

In pteropods, the precipitation of calcium carbonate mostly occurs at the shell edge [e.g. 28]. Even if precipitation takes place at low Ω_a_ value, shell dissolution probably still occurs elsewhere. Orr et al. [12] have demonstrated that pteropods were able to survive for 2 days in water undersaturated with respect to aragonite but their shells exhibited dissolution marks. Therefore, it is not certain whether pteropods can achieve a positive balance between CaCO_3_ precipitation and dissolution in undersaturated sea water. For these reasons, ^45^Ca needs to be interpreted with caution when organisms are subject to dissolution, as it does not enable to estimate the simultaneous dissolution of the shell. Future studies should aim at quantifying CaCO_3_ dissolution. The alkalinity anomaly technique [40] or changes in shell mass could be helpful but their use might be difficult due to the high sensitivity required to measure the small rate of CaCO_3_ precipitation and dissolution that prevail in pteropods. It is also important to design techniques which would enable to maintain pteropods for extended periods of time in the laboratory as longer perturbation experiments would likely allow to investigate whether pteropods can acclimate to corrosive waters.

In conclusion, this study is the first to provide information on the physiological response of a pteropod to levels of pCO_2_ and temperatures expected in the near future. The results on the size-weight relationship as well as on rates of respiration, gut clearance and calcium carbonate precipitation are critical information required to predict, through individual based models, the response of pteropods to the future environmental conditions. The logarithmic decrease of CaCO_3_ precipitation as a function of the aragonite saturation state and the fact that pteropods are able to precipitate calcium carbonate in undersaturated waters are particularly important parameters that will be useful in the global modeling on the impacts of ocean acidification on pelagic calcifiers.

## Materials and Methods

### Ethics Statement

This work has been conducted according to relevant national and international guidelines for ethics and animal welfare which do not include any specific requirement for planktonic mollusks.

### Sampling and experimental set-up

Sampling was undertaken in Kongsfjorden (Svalbard) during the period 22 May to 6 June 2009 Kongsfjorden is an open fjord, with a maximum depth of 250 m, and is influenced by both Atlantic and Arctic waters [41]. Pteropods were collected using a plankton net (modified WP2 net with 57 cm mouth diameter and 200 µm mesh size) that was gently towed during 2 to 4 min at 15 m depth. A purpose-designed collector with a large 5 l container was used to avoid any damage to the body or shell and minimize stress. After collection, the pteropods were immediately transported to the Kings Bay Marine Laboratory at Ny-Ålesund and maintained in. 20 l beakers under controlled conditions. The seawater used in the experiments was pumped at 80 m and filtered through 20 µm filters. A continuous pH-stat system (IKS, Karlsbad) that bubbled either CO_2_-free air or pure CO_2_ was used to control pH. For the sake of simplicity, the experimental conditions are named by the temperature index CT for the control temperature (∼0°C) and HT for the high temperature (∼4°C) followed by the approximate pCO_2_ values (280, 380, 550, 760 and 1120 µatm).

### Measurement of the carbonate chemistry

Seawater pH was measured daily in the experimental beakers and twice on surface (0.5 m) field samples, using a pH meter (Metrohm, 826 pH mobile) with a glass electrode (Metrohm, electrode plus) calibrated every second day on the total scale using Tris/HCl and 2-aminopyridine/HCl buffer solutions with a salinity of 35.0 [42]. Total alkalinity (*A*_T_) was measured in the experimental samples as well as on 8 surface field samples, which were filtered, poisoned with HgCl_2_ and analyzed within two days using a potentiometric titration and a Metrohm titrator (Titrando 80). Measurements were carried out on 25 ml samples at 21°C and *A*_T_ was calculated using a Gran function applied to the pH values ranging from 3.5 to 3.0 as described by Dickson et al. [42]. Titrations of a total alkalinity from standard seawater provided by A. G. Dickson (batch 90) were within 1.7 µmol kg^−1^ of the nominal value (standard deviation = 1.85 µmol kg^−1^; n = 12). The concentration of dissolved inorganic carbon (*C*_T_) was also measured on 6 surface field samples by using a AIRICA analyzer (Marianda, Kiel). *C*_T_ measurements were performed using 1200 µl samples. For calibration, 1100, 1200 and 1300 µl samples of the standard seawater (batch 90) were measured, a regression line drawn and the area for 1200 µl calculated using the regression parameters.

The correlation coefficients were typically >0.99. All the parameters of the carbon chemistry were determined from pH_T_ and *A*_T_ or *A*_T_ and *C*_T_, temperature and salinity using the R package seacarb [43].

### Size-weight relationship

The relationship between size and weight of pteropod was based on 70 organisms representative of the size range of the pteropods that were sampled in the Kongsfjorden during the study period. Shell sizes, based on the maximum diameter of the shell, were measured under a Leica binocular microscope. Pteropods were gently tissue-dried and weighted on a Mettler Toledo balance (±0.1 mg) and the following non-linear model was used to estimate their size- weight relationship:
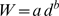
where *W* is the wet weight (including the shell), *a* is a constant, *d* is the shell diameter and *b* is the allometric coefficient.

### Respiration rate

A batch of 80 pteropods was separated into five equal groups and pre-acclimated during 24 h at the 5 pCO_2_ values. The same protocol was used for the control and high temperature conditions. After acclimation, 4 replicates were performed at each pCO_2_ level. Each replicate comprised three pteropods which were incubated in 60 ml bottles during 24 h. The initial and final oxygen concentrations were determined (see below). Furthermore, duplicates incubations without pteropods were also carried out in order to estimate the blank metabolism (i.e. microbial respiration). The oxygen concentration was determined on 50 ml subsamples using a Winkler titration with a 665 Dosimat titrator (Metrohm) fitted with a redox electrode (Metrohm, 6.0452.100). Pteropods were removed before the titration and the shell size was measured under the microscope as described above. The pteropod weight was determined from the size-weight relationship established previously.

### Gut clearance rate

Five groups of twenty freshly collected pteropods were incubated in the 5 pCO_2_ conditions at control temperature during 48 h in filtered (0.2 µm) sea water. Five replicates of single individuals were sampled at time points 0, 3, 9, 18 and 48 h. Another batch of 80 pteropods was divided in five equal parts and incubated in the different pCO_2_ conditions at elevated temperature during 72 h in filtered (0.2 µm) sea water. Four replicates of single organisms were sampled at time points 0, 4, 8, 20 and 72 h. Individuals were weighted and kept at −80°C pending their shipment in dry ice to the Villefranche Laboratory, France. Pigments were extracted by crushing the samples in 90% acetone (GR for analysis, Merck). The fluorescence of chlorophyll and phaeopigments was measured on a fluorometer (Tuner Designed) before and after acidification (50 µl of 0.3N HCl). A model of exponential reduction in gut fluorescence over time [44], was used to describe the decrease of the total content of chlorophyll and phaeopigments per mg of pteropod wet weight as a function of time:
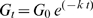
where *G_t_* and *G_0_* are the gut content at time *t* and the initial gut content, both in µg (g wet weight)^−1^, and *k* (h^−1^) is the gut clearance rate.

### ^45^Ca uptake

Freshly collected pteropods were incubated in five 20 l beakers at the control pCO_2_ (380 µatm) and were gradually brought to the 5 desired pCO_2_ values within 2 to 4 h. After 24 h of pre-acclimation to the different pCO_2_ conditions, the pteropods were transferred to 5 l beakers at the same pCO_2_ and temperature levels. pH was maintained at the desired level by the pH-stat system described above. The beakers were then spiked with ^45^CaCl_2_ in order to reach an activity of 50 Bq ml^−1^. The 5 groups (one at each pCO_2_ level) comprised 45 animals each that served for time points 0, 4 and 8 h. The same protocol was used on pteropods maintained at the control and the high temperature.

Five animals were sampled in triplicate at times 0, 4 and 8 h, rinsed with unlabeled seawater, gently dried with a tissue to remove seawater, and weighed (±0.1 mg). Shells were dissolved with 0.5N HCl in the counting vials and soft tissues were removed. The solution was then neutralized using 2N NaOH and 10 ml of scintillation liquid (Ultima Gold, Perkin Elmer) added. Counting of radioactivity was performed with a Packard scintillation counter. An identical protocol was used in the same conditions on pteropods killed by HgCl**_2_** prior to incubation in order to estimate the non-biological incorporation of ^45^Ca in the shell. The amount of CaCO**_3_** incorporated in the shell was calculated as described by Comeau et al. [28]

## Supporting Information

Figure S1 Size-wet weight relationship established on 70 individuals collected in the Kongsfjorden in May–June 2009. The regession is shown (y = 0.24×^3.04^).
(0.15 MB TIF)

Table S1 Mean seawater carbonate chemistry of surface seawater. The bold values correspond to the carbonate chemistry parameters measured. The partial pressure of CO_2_ (pCO_2_) and the saturation state of aragonite (Ω_a_) were derived from these values, the salinity (S) and the temperature (T).
(0.04 MB DOC)

Table S2 Mean seawater carbonate chemistry measured during the gut clearance and respiration rates experiments. The partial pressure of CO_2_ (pCO_2_) and the saturation state of aragonite (Ω_a_) were derived from pH_T_, total alkalinity (A_T_), salinity (S) and temperature (T).
(0.04 MB DOC)

Table S3 Mean seawater carbonate chemistry measured during the 45Ca experiments. The partial pressure of CO_2_ (pCO_2_) and the saturation state of aragonite (Ω_a_) were derived from pH_T_, total alkalinity (A_T_), salinity (S) and temperature (T).
(0.04 MB DOC)

Table S4 Respiration rates as a function of the incubation conditions (mean ± SD, n = 20).
(0.03 MB DOC)

Table S5 CaCO_3_ precipitation in the dead pteropods and net precipitation in the pteropods alive (mean ± SD, n = 20).
(0.03 MB DOC)
