# Supplementary figures and images for: Response of the Arctic Pteropod Limacina helicina to Projected Future Environmental Conditions
Source: PLoS One. 2010 Jun 29;5(6):e11362. doi: 10.1371/journal.pone.0011362 (PMC2894046; doi:10.1371/journal.pone.0011362)

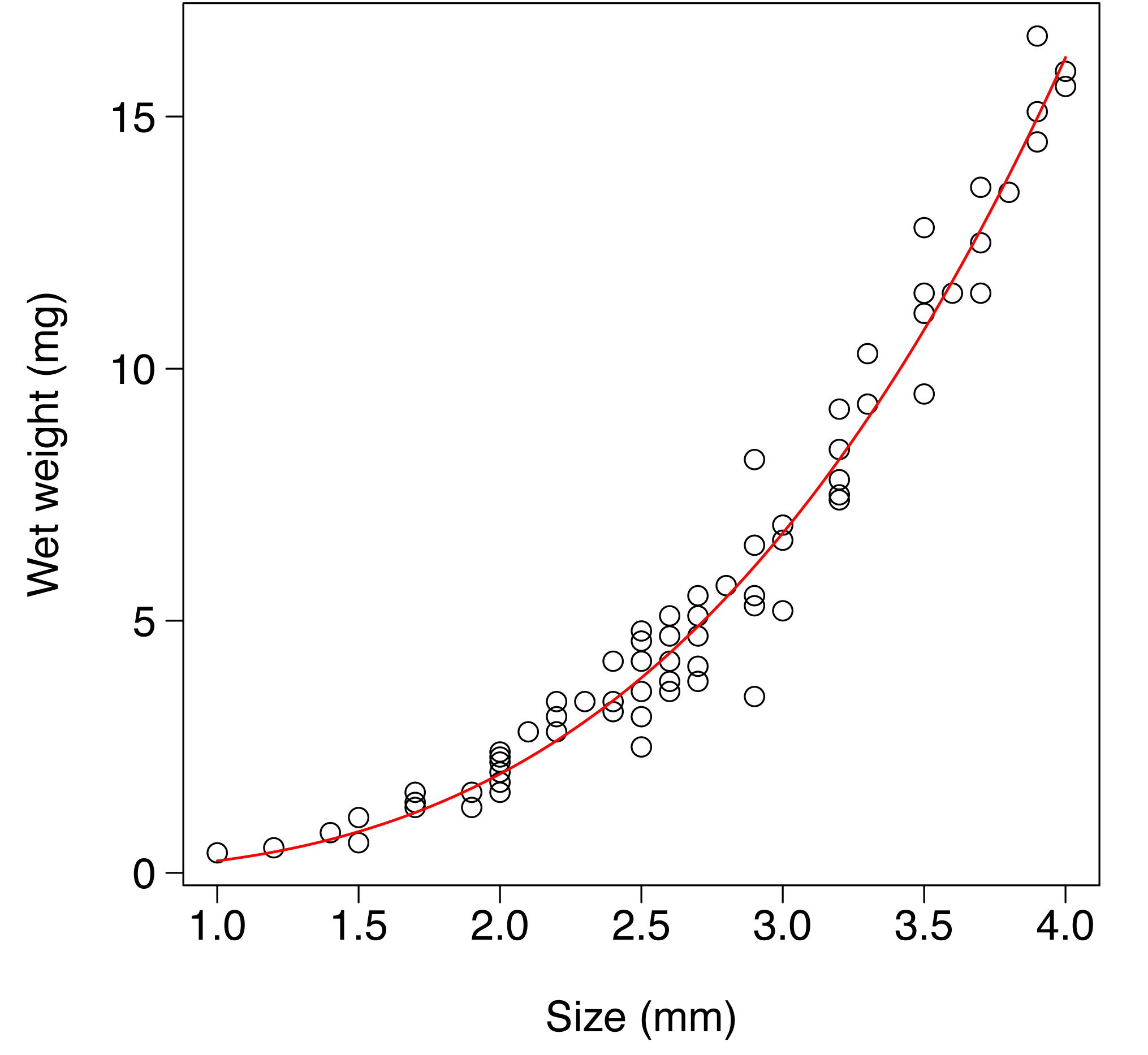

Supplement: Figure S1 — Size-wet weight relationship established on 70 individuals collected in the Kongsfjorden in May–June 2009. The regession is shown (y = 0.24×3.04). (0.15 MB TIF) [file pone.0011362.s001.tif]
