# Supplementary material for: Response of the Arctic Pteropod Limacina helicina to Projected Future Environmental Conditions
Source: PLoS One. 2010 Jun 29;5(6):e11362. doi: 10.1371/journal.pone.0011362 (PMC2894046; doi:10.1371/journal.pone.0011362)
Supplement: Table S1 — Mean seawater carbonate chemistry of surface seawater. The bold values correspond to the carbonate chemistry parameters measured. The partial pressure of CO2 (pCO2) and the saturation state of aragonite (Ωa) were derived from these values, the salinity (S) and the temperature (T). (0.04 MB DOC) [file pone.0011362.s002.doc]

| **Sampling date** | **pH_T_** | ***A*_T_**  (μmol kg^-1^) | ***C*_T_**  (μmol kg^-1^) | **pCO_2_**  (μatm) | **Ω_a_** | ***S*** | ***T***  (°C) |
| --- | --- | --- | --- | --- | --- | --- | --- |
| 2009-05-17 | 8.36 | 2277 | 2034 | 169 | 2.5 | 34.5 | -0.8 |
| 2009-05-17 | 8.30 | 2269 | 2053 | 194 | 2.42 | 34.5 | -0.8 |
| 2009-05-19 | 8.34 | 2275 | 2038 | 178 | 2.45 | 34.4 | -0.2 |
| 2009-05-19 | 8.32 | 2273 | 2044 | 186 | 2.37 | 34.4 | -0.2 |
| 2009-05-29 | 8.28 | 2275 | 2068 | 210 | 2.17 | 34.5 | -0.3 |
| 2009-05-29 | 8.25 | 2276 | 2080 | 225 | 2.07 | 34.5 | -0.3 |
| 2009-06-02 | 8.22 | 2273 | 2089 | 244 | 1.96 | 34.5 | -0.1 |
| 2009-06-02 | 8.20 | 2269 | 2095 | 257 | 1.88 | 34.5 | -0.1 |
