# Supplementary material for: Response of the Arctic Pteropod Limacina helicina to Projected Future Environmental Conditions
Source: PLoS One. 2010 Jun 29;5(6):e11362. doi: 10.1371/journal.pone.0011362 (PMC2894046; doi:10.1371/journal.pone.0011362)
Supplement: Table S3 — Mean seawater carbonate chemistry measured during the 45Ca experiments. The partial pressure of CO2 (pCO2) and the saturation state of aragonite (Ωa) were derived from pHT, total alkalinity (AT), salinity (S) and temperature (T). (0.04 MB DOC) [file pone.0011362.s004.doc]

| **Condition** | **pH_T_** | ***A*_T_**  (μmol kg^-1^) | **pCO_2_**  (μatm) | **Ω_a_** | ***S*** | ***T***  (°C) |
| --- | --- | --- | --- | --- | --- | --- |
| 280 CT | 8.17 | 2271 | 278 | 1.81 | 34.5 | 0.5 |
| 380 CT | 8.03 | 2271 | 399 | 1.36 | 34.5 | 0.5 |
| 550 CT | 7.90 | 2271 | 552 | 1.04 | 34.5 | 0.5 |
| 780 CT | 7.77 | 2271 | 760 | 0.78 | 34.5 | 0.5 |
| 1120 CT | 7.63 | 2271 | 1067 | 0.58 | 34.5 | 0.5 |
| 280 HT | 8.18 | 2279 | 267 | 2.13 | 34.5 | 3.9 |
| 380 HT | 8.06 | 2279 | 376 | 1.64 | 34.5 | 3.9 |
| 550 HT | 7.89 | 2279 | 578 | 1.16 | 34.5 | 3.9 |
| 780 HT | 7.80 | 2279 | 722 | 0.96 | 34.5 | 3.9 |
| 1120 HT | 7.61 | 2279 | 1148 | 0.63 | 34.5 | 3.9 |
