# Supplementary material for: Response of the Arctic Pteropod Limacina helicina to Projected Future Environmental Conditions
Source: PLoS One. 2010 Jun 29;5(6):e11362. doi: 10.1371/journal.pone.0011362 (PMC2894046; doi:10.1371/journal.pone.0011362)
Supplement: Table S4 — Respiration rates as a function of the incubation conditions (mean ± SD, n = 20). (0.03 MB DOC) [file pone.0011362.s005.doc]

| **Condition** | **Respiration rates**  **(**μmol O_2_ (g w w)^-1^h^-1^) | **Condition** | **Respiration rates**  **(**μmol O_2_ (g w w)^-1^h^-1^) |
| --- | --- | --- | --- |
| CT 280 | 5.6 ± 0.7 | HT 280 | 6.0 ± 0.5 |
| CT 380 | 5.7 ± 0.5 | HT 380 | 6.5 ± 0.5 |
| CT 550 | 5.9 ± 0.5 | HT 550 | 7.2 ± 0.7 |
| CT 760 | 5.9 ± 0.8 | HT 760 | 7.2 ± 0.4 |
| CT 1120 | 6.2 ± 0.3 | HT 1120 | 7.5 ± 0.6 |
