# Supplementary material for: Response of the Arctic Pteropod Limacina helicina to Projected Future Environmental Conditions
Source: PLoS One. 2010 Jun 29;5(6):e11362. doi: 10.1371/journal.pone.0011362 (PMC2894046; doi:10.1371/journal.pone.0011362)
Supplement: Table S5 — CaCO3 precipitation in the dead pteropods and net precipitation in the pteropods alive (mean ± SD, n = 20). (0.03 MB DOC) [file pone.0011362.s006.doc]

| **Condition** | **CaCO_3_ passive adsorbtion (dead pteropods)**  μmol (g w w)^-1^ h^-1^ | **CaCO_3_ precipitated**  μmol (g w w)^-1^ h^-1^ |
| --- | --- | --- |
| CT 280 | 0.30 ± 0.08 | 0.61 ± 0.05 |
| CT 380 | 0.32 ± 0.08 | 0.41 ± 0.04 |
| CT 550 | 0.37 ± 0.13 | 0.25 ± 0.04 |
| CT 760 | 0.36 ± 0.14 | 0.02 ± 0.04 |
| CT 1120 | 0.39 ± 0.08 | -0.05 ± 0.02 |
| HT 280 | 0.32 ± 0.04 | 0.68 ± 0.03 |
| HT 380 | 0.30 ± 0.07 | 0.46 ± 0.05 |
| HT 550 | 0.34 ± 0.10 | 0.44 ± 0.06 |
| HT 760 | 0.37 ± 0.09 | 0.25 ± 0.06 |
| HT 1120 | 0.37 ± 0.08 | 0.02 ± 0.04 |
